# Improving Ultrasound B-Mode Image Quality with Coherent Plane-Wave Compounding Using Adaptive Beamformers Based on Minimum Variance

**DOI:** 10.3390/s25051306

**Published:** 2025-02-21

**Authors:** Larissa C. Neves, Felipe M. Ribas, Joaquim M. Maia, Acacio J. Zimbico, Amauri A. Assef, Eduardo T. Costa

**Affiliations:** 1 Graduate Program in Electrical and Computer Engineering (CPGEI), Federal University of Technology—Paraná (UTFPR), Curitiba 80230-901, PR, Brazil; ribasf@alunos.utfpr.edu.br (F.M.R.); joaquim@utfpr.edu.br (J.M.M.); amauriassef@utfpr.edu.br (A.A.A.); 2 Department of Electrical Engineering (DEEL), Eduardo Mondlane University (UEM), Maputo 3453, Mozambique; ajzimbico@gmail.com; 3 Department of Electronics and Biomedical Engineering (DEEB) & Biomedical Engineering Centre (CEB), University of Campinas (UNICAMP), Campinas 13083-970, SP, Brazil; educosta@unicamp.br

**Keywords:** adaptive filters, beamforming, eigenspace, generalized sidelobe canceler, plane-wave, ultrasound images

## Abstract

Medical ultrasound imaging using coherent plane-wave compounding (CPWC) for higher frame-rate applications has generated considerable interest in the research community. The adaptive Eigenspace Beamformer technique combined with a Generalized Sidelobe Canceler (GSC) provides noise and interference reduction in images, improving resolution and contrast compared to basic methods: Delay and Sum (DAS) and Minimum Variance (MV). Different filtering approaches are applied in ultrasound image processing to reduce speckle signals. This work introduces the combination of beamformer Eigenspace Based on Minimum Variance (ESBMV) associated with GSC (EGSC) and the Kuan (EGSCK), Lee (EGSCL), and Wiener (EGSCW) filters and their enhanced versions to obtain better quality of plane-wave ultrasound images. The EGSCK technique did not present significant improvements compared to other methods. However, the EGSC with enhanced Kuan (EGSCKe) showed a remarkable reduction in geometric distortion, i.e., 0.13 mm (35%) and 0.49 mm (67%) compared to the EGSC and DAS techniques, respectively. The EGSC with Enhanced Wiener (EGSCWe) showed the best improvements in contrast radio (CR) aspects, i.e., 74% compared to the DAS technique and 60% to the EGSC technique. Furthermore, our proposed method reduces geometric distortion, making it a good option for plane-wave ultrasound imaging.

## 1. Introduction

In ultrasound imaging, beamforming is typically achieved using an array of transducers, where each transducer element acts as both an emitter and a receiver of ultrasonic waves. Multiple transducers are arranged in a specific configuration (e.g., linear, phased, or curvilinear) to allow control of the direction of the emitted beams. The signals from each transducer are then processed using beamforming algorithms, such as coherent plane-wave compounding (CPWC) [1,2].

The advantages of using CPWC over conventional radio frequency (RF) signals are primarily related to efficiency and image quality. Unlike RF-based beamforming, which often requires multiple signal emissions and complex processing to focus on specific points, plane waves allow simultaneous activation of all transducers in the array, covering a larger area in a single emission. This leads to faster data acquisition with fewer transmissions. Additionally, CPWC for ultrasound imaging can increase the framerate by more than 100 times that of conventional methods [3,4], improving image quality with dynamic focusing by combining multiple images acquired from different angles, which helps reduce artifacts and enhance tissue visualization [5]. This is possible because the number of scanlines no longer limits the framerate [3].

The increase in framerate allows for the analysis of structures that present movement, such as studies of blood flow [5], movement of tissues through shear waves [6], and real-time images [7], which helps in various areas of diagnosis.

Besides its application in ultrasound imaging, adaptive beamforming techniques are also widely used in communication systems to improve the performance of covert communication, ensuring greater directionality and security in the transmissions [8], or using beamforming in dynamic metasurface antennas (DMA) to optimize energy efficiency in multi-user systems [9]. Although the contexts are different, both studies share the goal of improving signal quality and reducing interference. Just as in ultrasound imaging, where adaptive beamforming is used to improve resolution and reduce speckle noise, beamforming techniques in communication systems aim to maximize signal efficiency and quality by using adaptive optimization to adjust the beamformer weights.

Delay and Sum (DAS) is the pattern method for generating plane-wave ultrasound imaging [3]. It is a non-adaptive beamforming technique in which the predefined and fixed weight applied to the signal is independent of the received signal. Although the DAS method could generate plane-wave ultrasound imaging with superior quality compared to some conventional imaging approaches using a focused beamformer [10], DAS still presents poor contrast and low image resolution [11].

Capon proposed an adaptive beamformer approach to improve the DAS technique [12]. It is based on finding a weighting factor that represents the signals of interest and minimizes the contribution of interference signals, analyzing the output power of the adaptive beamformer. This method is termed Minimum Variance (MV).

In the MV, as in the DAS method, the received echo signals are already delayed in time and are represented as the sum of two factors: the interfering signals and the echoes of the received signal. It is assumed that the interfering signals and the received echo do not correlate, so the applied weighting factor is determined by minimizing the signal output power [13].

Applebaum and Chapmam proposed an adaptive beamforming Generalized Sidelobe Canceller (GSC) [14], and its weighting factor is also calculated by minimizing the beamformer output power, but in terms of minimal linearly constrained variance [15]. The weight applied in the beamforming GSC is calculated by separating it into two orthogonal elements: non-adaptive and adaptive weights. Consequently, it is possible to suppress interference and noise present in the signal.

The adaptive beamforming Eigenspace Based on Minimum Variance (ESBMV) decomposes the covariance matrix (CVM) of the received signals into two subspace components, i.e., the noise subspace and the signal subspace. Therefore, the CVM is eigen decomposed in terms of the eigenvalues and the corresponding eigenvectors [13,16]. The more significant eigenvectors are related to the signal subspace, while the remaining are related to the noise subspace.

The eigenvectors associated with the larger eigenvalues are projected onto the MV weight vector to define the ESBMV weight vector. The ESBMV weight vector is designed to minimize the influence of sidelobe signals while preserving the desired signal [16].

Adaptive filters can remove unwanted elements from ultrasound images, reducing interference and noise signals. Speckle is one of the noise intrinsic to ultrasound images and decreases image resolution and contrast [17]. Different approaches, such as the Kuan, Lee, and Wiener filters, were adopted to reduce the speckle in the images [18].

This work proposes the implementation of the adaptive beamformer ESBMV in combination with the GSC method (EGSC), the Kuan (EGSCK), Lee (EGSCL), and Wiener filters, and their corresponding enhanced versions, i.e., the EGSCKe, the EGSCLe, and the EGSCWe beamformers to improve plane-wave ultrasound imaging.

## 2. Theoretical Basis of the Proposed Technique

For Minimum Variance beamformer, the output can be written as a vector of weights ($\vec{w}{\left(h\right)}^{H})$, applied in the received signals in the discrete time index (h), as presented in (1) [15], where the $X\left(h\right)$ is composed as the sum of the noise and interference signals (Ns(h)) and the received echo signal ($S\left(h\right)$), as in (2), where both have no correlation.

(1) $$Z\left(h\right)=\vec{w}{\left(h\right)}^{H}X\left(h\right),$$

(2) $$X\left(h\right)=S\left(h\right)+Ns\left(h\right)$$

In (1) and (2), $\vec{w}\left(h\right)={\;[w_{1}(h);\;w_{2}(h);\;\ldots \;;\;w_{E}(h)]}^{T}$ is a complex vector of weights, $X\left(h\right)={\left[x_{1}(h);\;x_{2}(h);\;\ldots \;;\;x_{E}(h)\right]}^{T}$ is the time-delayed version of array received signals, where *E* represents the total number of transducer elements, and ( )*^H^* is the Hermitian Matrix [15].

In the MV method, it is considered that the received signals in the direction of interest have no distortion, creating a constraint, which makes it possible to determine the weights by minimizing the output power (Pt) of the beamformer, as shown in (3) and (4).

(3) $$Pt=E\left\{{|Z}^{2}|\right\}={\vec{w}}^{H}Q\vec{w},$$

(4) $${\vec{w}}_{MV}=\arg\min{\vec{w}}^{H}Q\vec{w},\;subject\;to\;{\vec{w}}^{H}\vec{a}=1$$

The MV weight vector is calculated proportionally for each echo received, as in (5) [19], and it depends on the covariance matrix (CVM) *Q*, which estimates the correlation of the desired signal with the received signal and the steering vector, *a*, with 1 × E, used to represent the direction of arrival of a signal in relation to the transducer.

(5) $${\vec{w}}_{MV}=\frac{\;\vec{a}Q^{-1}}{{\vec{a}}^{H}Q^{-1}\vec{a}},$$

The CVM *Q* is given as in (6), where it is estimated considering the overlapped submatrices, *X_l_*, of the echo data. This estimation involves *A* data samples, equal to (2*K* + 1), and the value of *D*, equal to (*E* − *L* + 1), where *L* represents the predefined aperture determined by *L* ≤ *E*/2 [19].

(6) $$Q=\frac{\sum_{h=-K}^{K}\sum_{l=1}^{M-L+1}X_{l}\left(h\right)X_{l}{\left(h\right)}^{H}}{AD},$$

This work combines adaptive beamforming techniques with different filters to improve the ultrasound image quality (i.e., contrast ratio and spatial resolution). Among the adaptive methods, the ESBMV beamformer allows higher contrast ultrasound images than the MV method. Also, GSC beamforming [14] improves ultrasound image resolution, reducing the images sidelobes [15]. Thus, the implementation of the ESBMV combined with the GSC has been presented in [20], with noticeable improvements.

In the GSC beamformer [17], the GSC weight vector, ${\vec{w}}_{GSC}$, is defined by (7), combining the non-adaptive weight, Equation (8), and the adaptive weight, Equation (9), generating the output as in (10) [20]. This technique allows for reduction in the noise and interference signal of echo data.

(7) $${\vec{w}}_{GSC}={\vec{w}}_{q}-B{\vec{w}}_{a}$$

(8) $${\vec{w}}_{q}={\left(\vec{a}{\vec{a}}^{H}\right)}^{-1}\vec{a}$$

(9) $${\vec{w}}_{a}={\left(B^{H}QB\right)}^{-1}B^{H}Q{\vec{w}}_{q},$$

(10) $$Z_{GSC}\left(h\right)={\vec{w}}_{GSC}X\left(h\right),$$

The matrix, *B*, is an essential element for the GSC beamformer, where ${\vec{a}}^{H}B=0$, which filters the desired signals, allowing the application of the adaptive weight only to the signals corresponding to noise. The matrix, *B*, prevents the signals of interest from being canceled as interference [21].

Using the ESBMV beamforming, the CVM *Q* is constructed by the noise subspace ($E_{p}$) and the signal subspace ($E_{s}$), respectively, as in (11) [11].

(11) $$Q=U\Lambda U^{H}=E_{s}+E_{p},$$

The signal subspace, $E_{s}$, is defined using the eigenvectors (ν) that correspond to the largest eigenvalues (*λ*), where the energy for the main lobe signal component is present. These eigenvectors are obtained by applying the predetermined weight coefficient, δ, with the condition that *λ*_i_ ≥ *α λ*_1_ [20]. Thus, the signal subspace, $E_{s}$, and the ${\vec{w}}_{GSC}$ weight vector are combined to compute the ${\vec{w}}_{EGSC}$, the weight vector of the ESBMV with GSC (EGSC) beamforming, as in (12), generating the output, as in (13) [20].

(12) $${\vec{w}}_{EGSC}=E_{s}E_{s}^{H}{\vec{w}}_{GSC},$$

(13) $$Z_{EGSC}\left(h\right)={\vec{w}}_{EGSC}X\left(h\right),$$

Different filters can be applied to eliminate unwanted speckle noise in ultrasound images, which can degrade the image resolution and contrast ratio (CR) [17]. In this project, the Wiener, Lee, and Kuan filters, respectively, and their enhanced versions were applied to the corresponding weighting factors of the beamformers.

The Wiener filter is effective in improving image resolution, employing the lowest mean square error (MMSE) between the expected signal and the output power beamformer, described in Equation (14), where ${{\vec{w}}^{H}Q}_{n}\vec{w}$ represents the noise power of the output beamformer and $Q_{n}$ is the noise covariance matrix [18], producing a calculation of the output signal as shown in (15).

(14) $$H_{Wiener}=\frac{{\left|\;S\left(h\right)\;\right|}^{2}}{{\vec{w}}^{H}Q\vec{w}}=\frac{{\left|\;S\left(h\right)\;\right|}^{2}}{{{\left|\;S\left(h\right)\;\right|}^{2}+\vec{w}}^{H}Q_{n}\vec{w}},$$

(15) $$Z_{Wiener}\left(h\right)=H_{Wiener}\vec{w}{\left(h\right)}^{H}X\left(h\right),$$

Analyzing the noise power, a scaling factor (μ) can be applied to reduce its effects and generate a new beamformer output, as presented in (16), creating an enhanced Wiener filter and producing a new calculation of the output signal as shown in (17). In this work, the value defined for the scaling factor was μ = *L*, where *L* is the predefined aperture [11].

(16) $$H_{Wiener-e}=\frac{{\left|\;S\left(h\right)\;\right|}^{2}}{{\vec{w}}^{H}Q\vec{w}}=\frac{{\;|\;S\left(h\right)\;|}^{2}}{{{\left|\;S\left(h\right)\;\right|}^{2}+\mu \vec{w}}^{H}Q_{n}\vec{w}},$$

(17) $$Z_{Wiener-e}\left(h\right)=H_{Wiener-e}\vec{w}{\left(h\right)}^{H}X\left(h\right),$$

The adaptive filters developed by Lee and Kuan [22,23] utilize the local MMSE criterion to calculate an output signal, analyzing a window or region around the pixel being processed considering the local mean variance [24].

The output beamformer after application of the filters can be determined as in (18) and depends on the mean value of the window ($\bar{Im}$), the coefficient of the adaptive filter (F), and the value of the analyzed pixel ($I_{p}$). The coefficient $F_{Lee}$, by the Lee filter, and $F_{Kuan}$, by the Kuan filter, can be obtained using (19) and (20), respectively, where *C_n_* is the noise variation coefficient and *C_I_* is the coefficient of variation in the image with noise [24].

(18) $$Z_{Kuan\;/\;Lee}\left(h\right)=\bar{Im}\left(h\right)+F\left(h\right)\left(I_{p}\left(k\right)-\bar{Im}\left(h\right)\right),$$

(19) FKuanh=1−Cn2CI21+Cn2,

(20) $$F_{Lee}\left(h\right)=1-\frac{C_{n}^{2}}{C_{I}^{2}+C_{n}^{2}},$$

Applying the filters mentioned above to the EGSC beamformer, the coefficients of the Kuan and Lee filters (*F*) are calculated considering *C_I_* of the EGSC beamformer and *C_n_* of the EGSC***_n_*** beamformer (noise).

In ultrasound imaging, adaptive filters eliminate as much noise as possible, keeping relevant information in the images. The application and performance of filters will change depending on the signal strength in the analyzed region. It can be divided into three regions of signal strength where the filter application can be optimized. In regions with homogeneous signal strength, all values are close to each other, and the filter application will not generate a significant difference. However, it is more computationally costly, so an average of the intensities represents an unbiased estimate of the region [25]. The cutoff value for this class is given as *C_I_* ≤ *C_n_*.

Regions with points of interest, tissue textures, or edges are considered heterogeneous because they present different signal intensities. In these areas, filters often suppress information in addition to noise. Thus, two other conditions were created to improve the filtering process: when the filter application conditions are not satisfied, *C_I_* ≥ *C_max_*, and when they are satisfied, *C_n_* < *C_I_* < *C_max_*, with $C_{max}=\sqrt{3}C_{n}$ [25,26]. These conditions can be seen in (21).

(21) $$Z\left(h\right)=\left\{\begin{array}{cc} \bar{Im}\left(h\right), & C_{I}\leq C_{n}\\ \bar{Im}\left(h\right)+F\left(h\right)\left(I_{p}\left(h\right)-\bar{Im}\left(h\right)\right), & C_{n}<C_{I}<C_{max}\\ I_{p}\left(h\right), & C_{I}\geq C_{max} \end{array}\right.$$

## 3. Computer Modeling

This work uses the PICMUS (Plane Wave Challenge in Medical Ultrasound Imaging) [27] database, providing coherent plane-wave compounding (CPWC) datasets for simulation or from real data acquisition (obtained from the phantom model 040 GSE or in vivo) of spatial resolution and contrast.

The objective of the modeling was to simulate and evaluate the performance of the proposed techniques to improve the quality of the ultrasonic image. The modeling allowed for predicting how different approaches could affect the resolution, contrast, and accuracy of the obtained images, before being tested in real-world scenarios. Thus, the main focus was to validate the effectiveness of the techniques in a controlled environment, optimizing acquisition and processing parameters to ensure the best possible results in image quality.

All the corresponding data images consider the Verasonics Vantage 128 ultrasound equipment, manufactured by Verasonics in Kirkland, Washington, USA, and the data used consider the emission of 11 steered plane waves, with an angular step of 0.43° for a linear transducer (L11-4v) with 128 elements that operates at a center frequency of 5.21 MHz and 128 elements. Offline data processing was performed using the MATLAB R2021a software with the Field II [28] program with the support of the Ultrasound ToolBox (USTB) [29] to generate ultrasound imaging using the DAS and MV techniques.

The USTB tool was used as the basis for the DAS and MV method codes, and from this, the ESBMV, GSC, and EBGSC techniques were developed, both with and without the Lee, Kuan, and Wiener filters.

**Figure 1:**
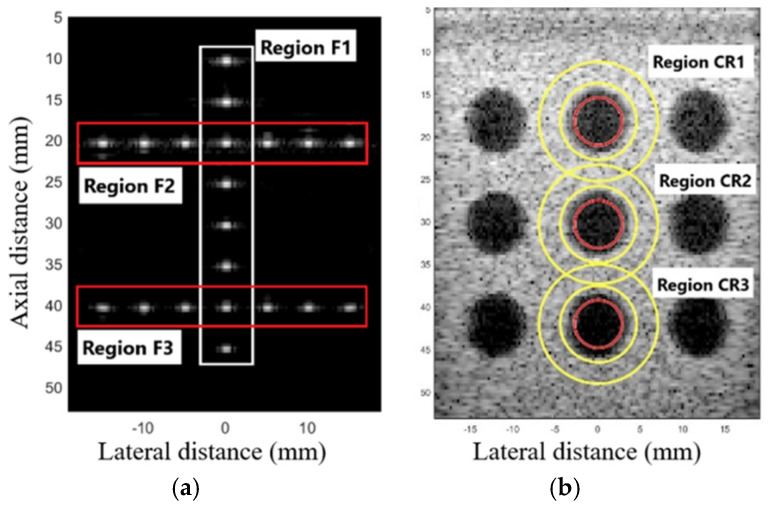
Selected regions for spatial resolution and contrast resolution evaluation. (**a**) Regions F1, F2, and F3 are used to calculate the mean FWHM*lat*. (**b**) Regions CR1, CR2, and CR3 for calculating the mean CR. The inner area of the red circle corresponds to the cyst area and the inner area between the yellow circles corresponds to the background area.

The parameter of the full width at half maximum (FWHM) was used to evaluate the geometric distortion in the F1, F2, and F3 regions, as shown in Figure 1a, measuring the FWHM in the axial (FWHM*Ax*) and lateral (FWHM*Lat*) axes. The average values of FWHM*Lat* were compared to the point of interest with an actual dimension of 0.10 mm in diameter [30]. For quantitative analysis, the contrast ratio (CR) was measured in the regions CR1, CR2, and CR3, as shown in Figure 1b, representing the difference between two areas: the mean amplitude of the phantom cyst (*φ_cyst_*) and the background (*φ_back_*), as presented in (22) [30].

(22) $$CR=\left|\varphi_{cyst}\right.-\left.\varphi_{bck}\right|,$$

## 4. In Situ Experiments

**Figure 2:**
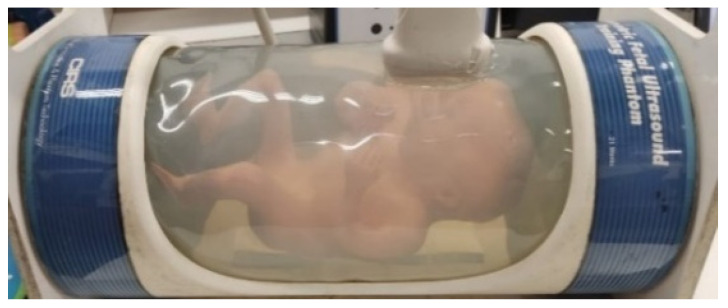
Fetal Ultrasound Biometrics mimicking the Phantom, CIRS 068 model.

**Figure 3:**
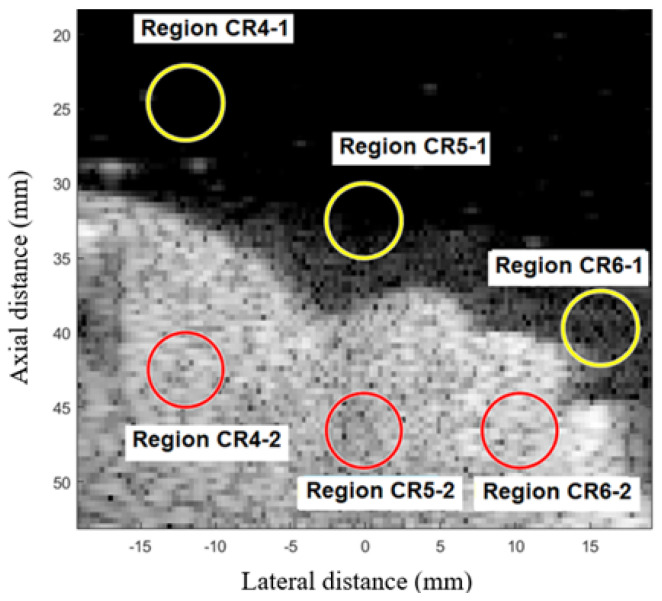
Regions CR4, CR5, and CR6 are used to calculate the average CR in fetal phantom images.

To evaluate contrast conditions, the proposed techniques were tested in the Fetal Ultrasound Biometrics Phantom (CIRS 068 model), Figure 2. Using this linear transducer, the range of the signal made it possible to visualize a region of the fetal head. The regions CR4, CR5, and CR6 were used to calculate the average CR in fetal phantom, as presented in Figure 3.

This acquisition was performed with the same configurations of the Verasonics Vantage 128 ultrasound equipment and processed using MATLAB, Field II, and USTB.

## 5. Results and Discussion

### 5.1. The Geometric Distortion

**Figure 4:**
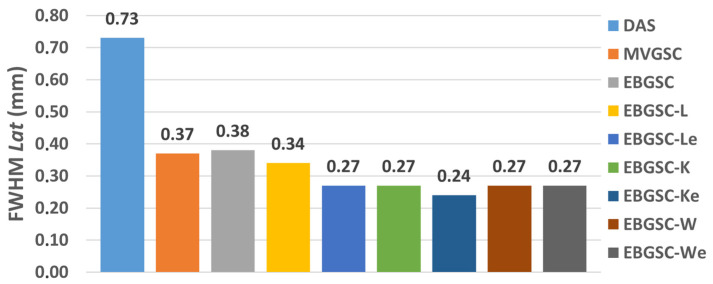
The simulation mean FWHM*Lat* values for DAS, GSC, EGSC, EGSCL, EGSCLe, EGSCK, EGSCKe, EGSCW, and EGSCWe, respectively.

The values of the FWHM on the lateral and axial axes are shown in Table 1 for the regions F1, F2, and F3, and Figure 4 shows the graph with the average values of the FWHM*Ax* and FWHM*Lat*.

**Figure 5:**
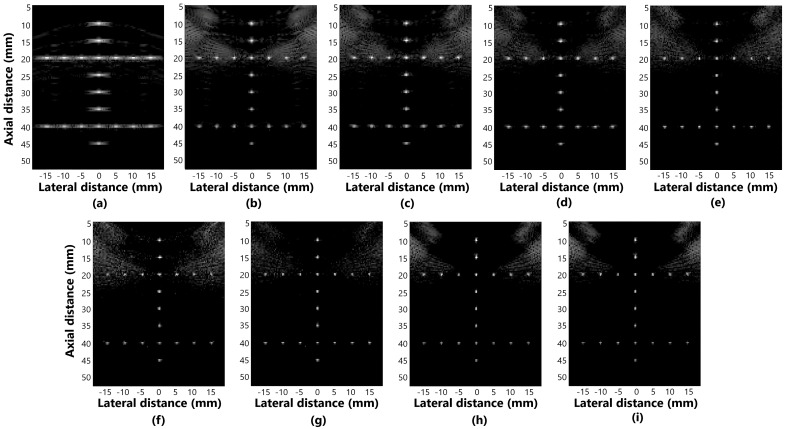
The displayed images for evaluating distortion in simulation response for (**a**) DAS, (**b**) GSC, (**c**) EGSC, (**d**) EGSCL, (**e**) EGSCLe, (**f**) EGSCK, (**g**) EGSCKe, (**h**) EGSCW, and (**i**) EGSCWe.

Figure 5a–i presents the beamformer simulation response for evaluating distortion in the methods: (a) DAS, (b) GSC, (c) EGSC, (d) EGSCL, (e) EGSC with the filter enhanced Lee (EGSCLe), (f) EGSCK, (g) EGSC with the filter enhanced Kuan (EGSCKe), (h) EGSCW, and (i) EGSC with the filter enhanced Wiener (EGSCWe).

We have noticed that, in the axial axis, the techniques presented relatively closer values of the FWHM, which means that there is no evident geometric distortion, as seen in Figure 5. In the lateral axis, more distortions occur, especially in the DAS method.

The EGSCW, EGSCWe, EGSCLe, and EGSCK equally decrease the average FWHM*Lat*, obtaining a reduction of 0.46 mm (63%), compared to the DAS method, with 0.10 mm (27%) and 0.09 mm (25%) compared to EGSC and GSC techniques, respectively.

The EGSCKe presented the result closest to the actual value, representing a reduction in the average FWHM*Lat* of 0.49 mm (67%), 0.13 mm (35%), and 0.12 mm (33%) compared to DAS, EGSC, and GSC techniques, respectively.

### 5.2. Contrast

**Figure 6:**
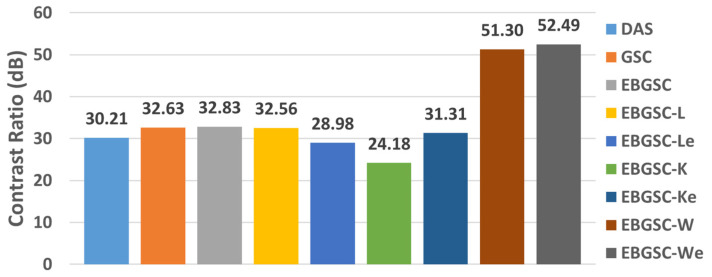
The simulation mean CR values for DAS, GSC, EGSC, EGSCL, EGSCLe, EGSCK, EGSCKe, EGSCW, and EGSCWe, respectively.

The values of CR are shown in Table 2 for the regions CR1, CR2, and CR3, and Figure 6 shows the graph with the average contrast for each method. The values in Figure 6 show that all the methods combined with the Wiener filter obtained better contrast values than the other techniques.

**Figure 7:**
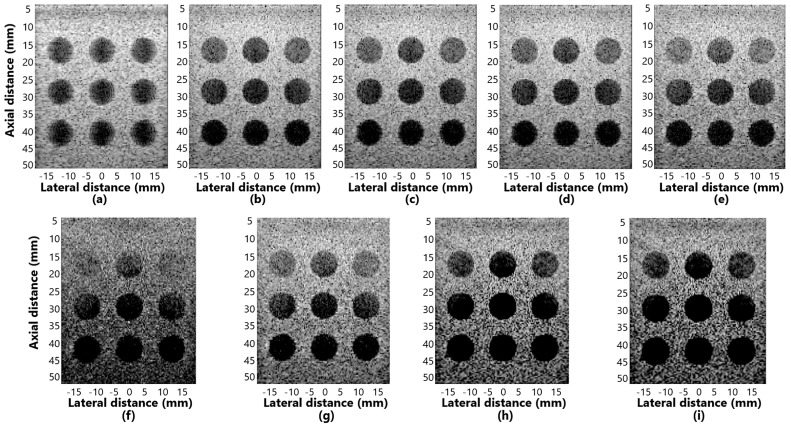
The displayed images for contrast analysis in simulation response for (**a**) DAS, (**b**) GSC, (**c**) EGSC, (**d**) EGSCL, (**e**) EGSCLe, (**f**) EGSCK, (**g**) EGSCKe, (**h**) EGSCW, and (**i**) EGSCWe.

Figure 7a–i shows the displayed images for simulation of contrast for the methods: (a) DAS, (b) GSC, (c) EGSC, (d) EGSCL, (e) EGSCLe, (f) EGSCK, (g) EGSCKe, (h) EGSCW, and (i) EGSCWe.

The EGSCWe technique generated a contrast mean value of 52.49 dB, representing an average contrast improvement of 19.66 dB (60%), 19.86 dB (61%), and 22.28 dB (74%) compared to EGSC, GSC, and DAS, respectively. We noticed that the EGSCWe improved the performance of the EGSCW contrast by 1.19 dB, approximately 2.3%.

The EGSCL shows a contrast value close to GSC and EGSC methods and presents an improvement of contrast of 2.35 dB (7.8%) with regard to the DAS technique. However, the EGSCLe decreased the contrast by 3.85 dB (11.7%), 3.65 dB (11.2%), and 1.23 dB (4%) compared to EGSC, GSC, and DAS, respectively.

The EGSCK technique presents the worst contrast value on average, with a reduction of 8.65 dB (26.3%), 8.45 dB (26%), and 6.03 dB (20%) compared to the EGSC, GSC, and DAS, respectively. Also, the EGSCKe shows a slight contrast improvement of 1.1 dB (3.6%) compared to the DAS method.

**Figure 8:**
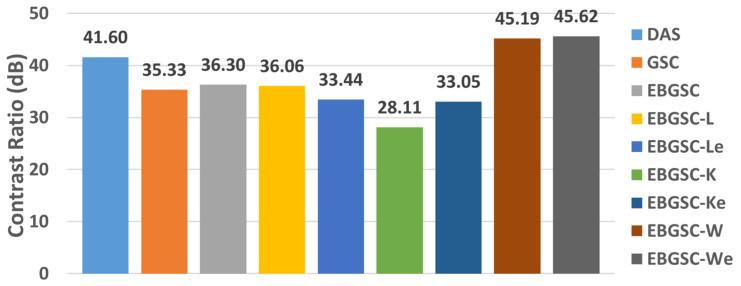
The fetal phantom mean CR values, for DAS, GSC, EGSC, EGSCL, EGSCLe, EGSCK, EGSCKe, EGSCW, and EGSCWe, respectively.

**Figure 9:**
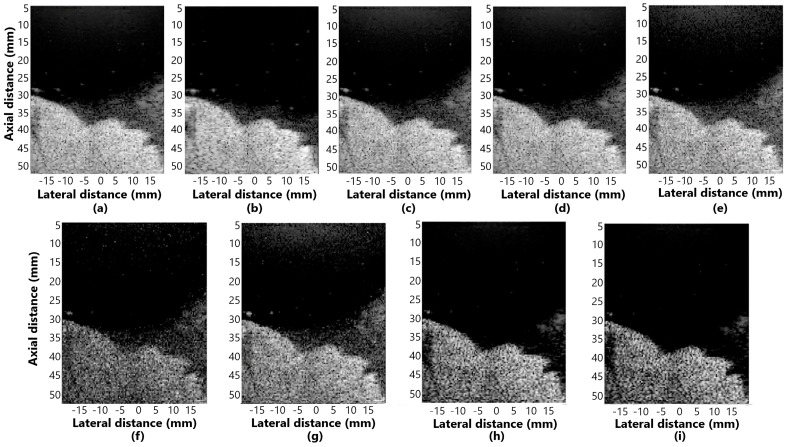
The displayed images for contrast analysis in fetal phantom for (**a**) DAS, (**b**) GSC, (**c**) EGSC, (**d**) EGSCL, (**e**) EGSCLe, (**f**) EGSCK, (**g**) EGSCKe, (**h**) EGSCW, and (**i**) EGSCWe.

Regions close to the contour of the fetal phantom face were analyzed, the average contrast for each method is presented in the graph of Figure 8 and Figure 9a–i shows the images displayed for simulation in fetal phantom for the methods: (a) DAS, (b) GSC, (c) EGSC, (d) EGSCL, (e) EGSCLe, (f) EGSCK, (g) EGSCKe, (h) EGSCW, and (i) EGSCWe.

The EGSCW and EGSCWe methods obtained a reduction in the noise around the fetus, providing better visualization of the contour.

Numerically, the contrast value increased with these techniques compared to the others. These methods showed improvement of 3.59 dB (9%) and 4.02 dB (10%), respectively, compared to the DAS method. An improvement, in contrast, allows better visualization of different areas, densities, and contours, being a positive result.

The comparison between the simulation and in situ experimental results reveals strong consistency, in terms of contrast. While simulations provide valuable theoretical data, the in situ experiments confirmed the effectiveness of techniques such as the EGSCWe and EGSCW, which improved image quality in both simulations and practical conditions. The ability to achieve similar improvements in both domains suggests that these methods are robust, highlighting the potential applicability of these methods in real clinical scenarios.

The geometric distortion results with the EGSCL and EGSCLe methods presented an improvement in the FWHM for the simulation and phantom, compared to EBGSC, GSC, and DAS. In the contrast results, for simulation and phantom images, the regions closest to the transducer suffer a slight homogenization due to filtering, which decreases contrast, especially for the EGSCLe. However, EGSCL and EGSCLe generally presented values around the MVGSC and EGSC methods, indicating future research with these filters.

The EGSCK and EGSCKe methods showed excellent FWHM reductions for simulation and phantom data compared to the DAS, MVGSC, EGSC, EGSCL, and EGSCLe methods. However, in terms of contrast, the EGSCKe method presented values close to the DAS, and the EGSCK presented a significant decrease with the lowest contrast values, which may indicate that there are better techniques to improve this parameter.

In research presented by Tasnim et al. [18], the Kuan and Lee filters were applied in post-processing B-Mode ultrasound images of different areas: kidney, fetus, cyst, and liver. Analyzing other measures, including signal-to-noise ratio (SNR), it was possible to verify that these filters improved or worsened the image depending on the area of the body analyzed, compared to basic methods such as the median filter.

Wu et al. [17] also used the Lee filter in kidney and liver images, and the Lee filter allowed an increase in the SNR compared to the median filtering technique. This shows us that the results depend on the methods and regions applied, indicating further research with the Lee and Kuan filters.

Aliabadi et al. [11], evaluating the ESBMV method with Wiener filter (ESBMVW) and improved Wiener filter (ESBMVWe), achieved CR increments of 46.3 dB (133%) and 26.1 dB (129%) for the ESBMVW, and 52.5 dB (164%) and 32.3 dB (160%), for the ESBMVWe, in comparison with to the DAS and MV techniques, respectively. The research was carried out with values of L = 48, a center frequency of 5 MHz, and a transducer with 128 elements.

Another research study using the ESBMVW, by Zhao et al. [31], evaluating targets of 0.1 mm in diameter, obtained significant FWHM reductions of 0.02 mm (50%) and 0.42 mm (95%) compared to the MV and DAS method. Evaluating the CR using the ESBMVW beamformer presented the same positive characteristic, generating improvements of 27.44 dB (239%) and 25.69 dB (194%) concerning the DAS and MV.

Compared to conventional methods, the improvement in contrast and reduction in distortion could represent better visualization of tissues, detection of small targets, and greater detail of the areas analyzed, which is positive for clinical use, helping with the quality of B-mode images and also in imaging modes that require high framerate, such as elastography, ultrafast Doppler, and ultrasound localization microscopy (ULM).

In ULM imaging, for example, which uses microbubbles to generate images of microvessels, high framerates are required to create images. However, there are limitations in spatial resolution due to the point spread functions (PSFs) of multiple microbubbles, which, despite developments in recent years, still makes obtaining high-quality images a challenge [32,33,34]. Thus, techniques such as those proposed in this work that reduce lateral distortion could improve spatial resolution, enabling the reduction in errors and time in detecting microbubbles, consequently, generating better images of small vessels.

The sizes of the signal subspace functions in the beamformer eigenspace and the windows for the adaptive filters can present different contrast and lateral distortion results, which can help analyze different regions and need to be better studied.

In this work, we did not do tests with 3D Ultrafast images. However, as the filtering techniques improved the resolution, they can be applied to each processed frame before reconstructing the high-resolution 3D images.

The results obtained in this work, along with those reported in [11,17,18,31], underscore the effectiveness of the EGSCW and EGSCWe methods in enhancing the contrast ratio and spatial resolution of ultrasound images. These findings highlight the potential for further research into these methods.

## 6. Conclusions

This work proposes a novel adaptive beamformer approach for medical ultrasound imaging with higher frame-rate applications using coherent plane-wave compounding (CPWC). Our proposed methodology, the ESBMV beamformer associated with GSC and advanced filtering techniques, improves the quality of plane-wave ultrasound images.

The EGSCWe and EGSCW beamformers obtained the best results compared to the ESBMV, GSC, and DAS techniques, reducing distortion and improving contrast. The proposed methods allow obtaining images with significant improvements compared to the ESBMV, GSC, and DAS methods. This suggests that the implemented techniques could generate higher-quality images using less processing time or acquire images with better quality and more details than the base techniques.

The proposed methods involving the EGSC with advanced filtering techniques are relatively new. Consequently, additional research is necessary to evaluate their potential for improving image quality and assisting in medical diagnosis.

## Figures and Tables

**Table 1:** Values of FWHM on the lateral and axial axis in the regions F1, F2, and F3 for the simulation image with FIELD II.

FWHM (mm)—FIELD II						
Beamformer	Axial			Lateral		
	F1	F2	F3	F1	F2	F3
DAS	0.28	0.25	0.29	0.73	0.66	0.79
GSC	0.30	0.32	0.35	0.34	0.34	0.41
EGSC	0.27	0.28	0.30	0.33	0.34	0.45
EGSCL	0.27	0.26	0.28	0.32	0.29	0.41
EGSCLe	0.27	0.24	0.24	0.26	0.22	0.32
EGSCK	0.25	0.22	0.25	0.30	0.23	0.28
EGSCKe	0.26	0.22	0.24	0.23	0.20	0.30
EGSCW	0.25	0.27	0.31	0.23	0.23	0.34
EGSCWe	0.26	0.25	0.31	0.24	0.22	0.34

**Table 2:** Values of contrast in the regions CR1, CR2, and CR3 for the simulation image with FIELDII.

CR (dB)—FIELD II			
Beamformer	CR1	CR2	CR3
DAS	29.32	30.38	30.92
GSC	30.38	32.45	35.06
EGSC	30.16	32.94	35.39
EGSCL	30.00	32.70	34.99
EGSCLe	24.26	30.10	32.60
EGSCK	14.32	26.75	31.47
EGSCKe	23.50	33.26	37.18
EGSCW	39.65	55.11	59.14
EGSCWe	40.69	56.34	60.46

## Data Availability

Dataset available on request from the authors.
